# Circulating Methemoblogin Fraction in Dogs With Sepsis

**DOI:** 10.3389/fvets.2020.00341

**Published:** 2020-06-16

**Authors:** Roberta Troia, Elena Ciuffoli, Kateryna Vasylyeva, Armando Foglia, Francesco Dondi, Massimo Giunti

**Affiliations:** Department of Veterinary Medical Sciences, Alma Mater Studiorum, University of Bologna, Bologna, Italy

**Keywords:** methemoglobin, CO-oximetry, sepsis, septic shock, canine, nitric oxide

## Abstract

Large amount of nitric oxide (NO) can be released in patients with sepsis. Methemoglobin is formed from the interaction between NO and hemoglobin. Mild methemoglobinemia reflecting NO overproduction has been reported in septic people, and occasionally associated to septic shock and organ dysfunction. The aim of this retrospective study was to evaluate circulating methemoglobin fraction in dogs with sepsis and to assess its prognostic value. Methemoglobin reference interval (RI) was calculated in 41 healthy dogs and was set at 0–2.2%. A total of 131 dogs with sepsis were included in the study; 24/131 had a circulating methemoglobin ≥2.2%. The median methemoglobin fraction was significantly higher in dogs with sepsis compared to healthy ones (1.7%, 0.4–3.5% vs. 1.0, 0.3–2.2%, *P* = 0.0005). No significant difference was observed between dogs with uncomplicated sepsis (*n* = 98) vs. dogs with septic shock (*n* = 33) (1.8%, 0.4–2.8% vs. 1.5%, 0.4–3.5%, *P* = 0.74), between dogs with and without multi-organ dysfunction (*n* = 38 and *n* = 93, respectively) (1.7%, 0.4–3.5% vs. 1.7%, 0.5–2.8%, *P* = 0.27), and between survivors (*n* = 77) vs. non survivors (*n* = 54) (1.5%, 0.4–2.8% vs. 1.8%, 0.4–3.5%, *P* = 0.05). Dogs with methemoglobin fraction above or equal to the upper limit of the RI had a significantly higher frequency of death compared to dogs with methemoglobin levels <2.2% (60.0% vs. 36.8%, *P* = 0.04). In conclusion, mild methemoglobinemia is detected in dogs with sepsis, and methemoglobin values above the RI might be associated with a worse outcome.

## Introduction

Methemoglobinemia describes a state where the iron component of heme within hemoglobin is oxidized from the ferrous to the ferric state. Methemoglobin is unable to bind oxygen, and its overproduction leads to impaired aerobic cellular metabolism, hypoxia, chocolate-brown colored blood and mucous membranes, cyanosis, and death (1–4). Low levels of methemoglobin are normally produced by auto-oxidation of hemoglobin *in vivo*. In steady state conditions, however, methemoglobin is rapidly recycled back to hemoglobin by methemoglobin-reducing enzymes, so that normal methemoglobin concentrations are usually kept <0.5–3% of total hemoglobin in humans and dogs (1, 3, 4). Both congenital and acquired forms of abnormal methemoglobinemia have been reported in human and veterinary literature (1, 2). In small animals, clinically significant methemoglobinemia usually arises from exposure to toxicants or chemicals able to induce hemoglobin oxidation (e.g., aniline, sulfonamides, nitrates, acetaminophen, hydroxicarbamide, tetracaine) (5–7). Congenital juvenile methemoglobinemia associated with

methemoglobin reductase deficiencies has also been described in several breeds of dogs, but it appears to be a rare disease (3, 4).

In humans, sepsis is considered a differential diagnosis among the endogenous causes of methemoglobinemia both in adult and pediatric patients (2, 8, 9). Inflammatory cytokines and bacterial lipopolysaccharides in sepsis activate endothelial cells and stimulate the production of an inducible form of nitric oxide synthase (iNOS) (2, 8, 10). As a result, nitric oxide (NO) is produced, contributing to vasodilation and distributive shock. NO acts as a cytostatic and cytotoxic molecule against microorganisms and host cells, and interacts with hemoglobin forming methemoglobin and nitrates. Nitrates can be converted into nitrite, and to further methemoglobin and NO in the presence of nitrate-producing bacteria through a vicious cycle (1, 2, 8, 10). Thus, the high methemoglobin levels reported in sepsis are thought to reflect such iNOS and NO overproduction, and might be a marker of sepsis severity (11). Additional causes of methemoglobinemia in sepsis include reduced activity of methemoglobin-reducing enzymes or lack of energy substrate for these enzymes (8). Risk factors or concurrent conditions such as anemia, acidosis, cardiocirculatory failure, dysbiosis, and slow intestinal transit may further contribute to superoxide radicals and NO formation, leading to methemoglobin overproduction (1, 2).

Higher methemoglobin levels were reported in septic compared to non-septic adult patients (8, 9). However, methemoglobin levels above 2% were rare during sepsis, and no association with outcome was identified (9). Similarly, comparable values of blood methemoglobin were reported between humans with sepsis and septic shock (8).

Methemoglobinemia can represent a significant underdiagnosed condition in septic neonates, because methemoglobin reducing-enzymes activities are decreased compared to adults, and because of the higher susceptibility of fetal hemoglobin to oxidative damage (2). In a study evaluating the occurrence and the risk factors for methemoglobinemia in a neonatal intensive care unit (ICU), patients with detectable methemoglobin were more frequently pre-term and showed a greater rate of culture-proven sepsis. The authors concluded that significant underdiagnosed methemoglobinemia is frequent in neonatal sepsis due to nitrate-producing bacteria (2). Methemoglobin and nitrite/nitrate values were greater in children with septic shock compared to healthy control patients; nonetheless, methemoglobin values were not correlated with scores of disease severity nor with any clinical variable of interest (10).

Few studies on NO, nitrites and nitrates have been performed in dogs. Although a causative role for NO and its metabolites has been suggested in the pathogenesis of sepsis-induced hypotension, results are controversial (12–14). To the best of the authors' knowledge, the occurrence and the clinical significance of abnormal circulating methemoglobin fraction as a marker of NO overproduction, have not been described in dogs with sepsis.

The aims of the current study were (1) to evaluate the prevalence of methemoglobinemia in dogs with sepsis and septic shock (2) to investigate for associations between circulating methemoglobin fraction and sepsis severity. We hypothesize that circulating methemoglobin fraction is higher in dogs with sepsis compared to healthy controls, and increase with sepsis severity and presence of septic shock.

## Materials and Methods

### Animals

A database of the medical records from dogs with sepsis hospitalized in the ICU of a Veterinary Teaching Hospital between November 2016 and November 2019 was retrospectively searched for cases with an available methemoglobin measurement at the time of admission. A proportion of the included dogs were part of a previous prospective study on sepsis and multiorgan dysfunction syndrome (MODS) approved by the local Institutional Animal Care and Use Committee.

Dogs were diagnosed with sepsis if at least 2/4 systemic inflammatory response syndrome (SIRS) criteria were satisfied, as previously reported (15), and an infection was confirmed by means of cytology, microbiology, histopathology or real-time polymerase chain reaction. Venous methemoglobin fraction assessed at the time of hospital admission had to be available for inclusion in the study.

Dogs with a history of prior exposure to toxicants, chemicals or drugs known to promote abnormal methemoglobinemia (e.g., acetaminophen, nitrates, sulfonamides, local anesthetics) were excluded from the study.

Forty-one healthy dogs were enrolled to determine reference intervals (RI) for venous blood gas variables and hemoglobin fractions, including methemoglobin. These were privately-owned blood donor dogs or staff-owned dogs with no history of recent or chronic medical conditions, classified healthy on the basis of history, physical examination, complete blood count (CBC), serum chemistry, blood gas analysis and urinalysis results. Inclusion of healthy and septic dogs was approved by the local Institutional Animal Care and Use Committee (ID 747 and 846, respectively).

### Data Collection

Various patient parameters were recorded at the time of admission, including body weight and physical examination findings. Data of venous blood gas analysis and methemoglobin fraction were determined by CO-oximetry using a blood gas analyzer (ABL 800 FLEX, Radiometer Medical ApS, Copenhagen, Denmark). Results of the blood gas analysis were corrected to the temperature of the patients. Hemoglobin fractions measurement was based on an optical assay using a 128-wavelenght spectrophotometer that was routinely calibrated and controlled on a weekly basis (ctHb calibration solution S7770 and AutoCheck quality control system solution, Radiometer Medical ApS, Copenhagen, Denmark). For blood gas analysis and methemoglobin measurement, venous blood samples were collected in lyophilized lithium heparin syringes (S-Sarstedt Monovette 1.2 ml LH) from jugular or saphenous venipuncture, sealed anaerobically and analyzed immediately. Additional laboratory data included a CBC assessed by an automated hematology system (ADVIA 2120, Siemens Healthcare Diagnostics, Erlangen, Germany) combined with microscopical blood smear evaluation, chemistry profile including measurement of serum creatinine, total bilirubin, albumin and C-reactive protein (CRP) concentration (AU 480, Beckman Coulter-Olympus, Brea, California, USA), coagulation profile including prothrombin time and partial thromboplastin time (BFT II, Siemens, Munich, Germany).

### Patient Grouping and Severity Scores

Dogs were grouped based on the presence of sepsis and septic shock. Septic shock was defined as the presence of hypotension (systolic blood pressure <90 mm Hg) (petMAP graphic, Ramsey Medical Inc., Tampa, FL) in a euvolemic patient and/or persistent hyperlactatemia despite fluid resuscitation (16).

When available, the fast Acute Patient Physiologic and Laboratory Evaluation (APPLE_fast_) score was calculated according to Hayes et al. (17), and used to group dogs (APPLE_fast_ ≥ 25 and <25) based on the cut-off with the highest specificity for death prediction in that cited study (17).

Organ dysfunction criteria were adapted from available canine literature as previously reported (15), and MODS was defined as the presence of at least two dysfunctional organs other than the one involved in the septic process.

Outcome was recorded as survival to hospital discharge, death, or euthanasia for ethical reasons due to moribund disease. Dogs euthanized for financial reasons were excluded from the study.

## Statistical Analysis

Data were expressed by standard descriptive statistics and presented as mean ± standard deviation or median and range (min-max), based on their distribution. Normality was assessed graphically and by using the D'Agostino-Pearson test. The RI for venous methemoglobin fraction was calculated using the Robust method with 90% CI of the reference limit, considering a right sided distribution. The Mann–Whitney *U*-test and the Kruskall–Wallis test with compensated *post-hoc* analysis were used to evaluate differences between groups. Categorical variables were compared using the Fisher test. Scatterplots and calculation of Spearman's correlation coefficients were used to assess correlations between continuous variables. Results were considered significant for a P < 0.05. Statistical analyses were performed using an online available statistical software (MedCalc Statistical Software version 18.10.2; MedCalc Software bvba, Ostend, Belgium).

## Results

A total of 131 dogs with sepsis were enrolled in the present study. Median age was 8.0 years (range 0.1–16 years), and median body weight was 13.2 kg (range 1.5–59.8 kg). Sex distribution was as follow: 41/131 (31%) intact males, 13/131 (10%) castrated males, 51/131 (39%) intact females, and 26/131 (20%) spayed females. Mixed-breed dogs were 39/131 (30%), while purebred dogs were 92/131 (70%). Median length of hospitalization was 3 days (0–19 days). Ninety-eight dogs out of 131 were diagnosed with sepsis (75%), while 33/131 (25%) had septic shock. Causes of sepsis in the whole population included pyometra (*n* = 31), septic peritonitis (*n* = 28), parvoviral enteritis (*n* = 19), pneumonia (*n* = 14), bite wounds (*n* = 8), bacterial prostatitis (*n* = 8), pyelonephritis (*n* = 5), necrotizing fasciitis (*n* = 4), cholangitis (*n* = 4), pancreatic abscess (*n* = 3), bacterial endocarditis (*n* = 3), penetrating trauma (limb *n* = 1; pelvic *n* = 1), perineal abscess (*n* = 1), bacterial lymphadenitis (*n* = 1). The occurrence of selected organ dysfunctions in the dogs enrolled was as follows: 36/131 (28%) renal, 25/131 (19%) cardiocirculatory, 23/131 (18%) hemostatic, 21/131 (16%) hepatic, 18/131 (14%) respiratory. Multiorgan dysfunction syndrome, as previously defined, was diagnosed in 38/131 (29%) dogs. Overall, 77/131 (59%) dogs survived to hospital discharge, while 54/131 (41%) were non-survivors.

The 41 healthy control dogs had a median age of 3.9 years (range 1.0–10.8 years), and a median body weight of 20.8 kg (range 5.3–43.8 kg). There were 14/41 (34%) intact females, 11/41 (27%) spayed females, 9/41 (22%) intact males and 7/41 (17%) castrated males. Mixed breed dogs were 26/41 (63%), while pure-breed dogs were 15/41 (37%) (2 Lagotto Romagnolo, 1 Dobermann, 1 French Bulldog, 1 Jack Russel Terrier, 1 Golden Retriever, 1 Galgo Espanol, 1 Cocker Spaniel, 1 Australian Shepherd, 1 Rhodesian ridgeback, 1 Alaskan Malamute, 1 Hovawart, 1 Labrador Retriever, 1 Border Collie, 1 German Shepherd). Median methemoglobin value in healthy dogs was 1% (range 0.3–2.2%). The RI for venous methemoglobin fraction was set at 0–2.2% (CI 1.8–2.5%).

**Figure 1:**
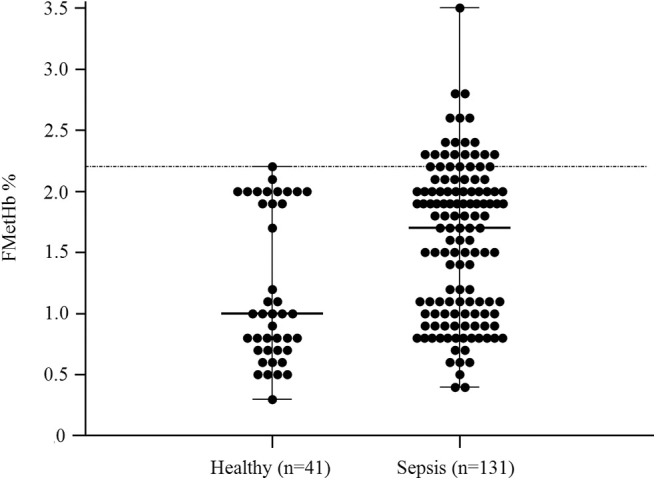
Dot plots for methemoglobin fraction (FMetHb %) in healthy control dogs compared to dogs with sepsis. The horizontal bars represent the median (central bar), and the minimum and maximum values (peripheral bands). The dotted line marks the upper reference interval for venous methemoglobin (2.2%). The difference was statistically significant (*P* = 0.007).

**Figure 2:**
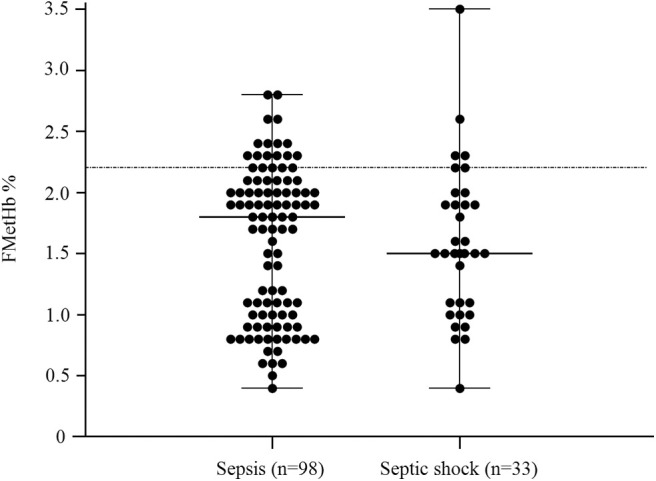
Dot plots for methemoglobin fraction (FMetHb %) in dogs with sepsis compared to dogs with septic shock (*P* = 0.74). The horizontal bars represent the median (central bar), and the minimum and maximum values (peripheral bands). The dotted line marks the upper reference interval for venous methemoglobin (2.2%).

**Figure 3:**
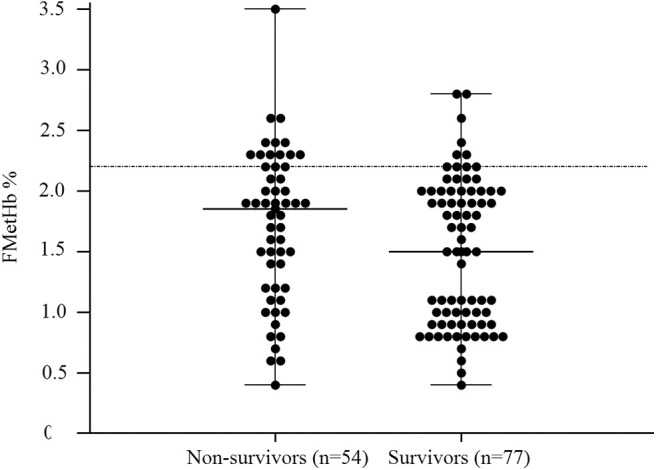
Dot plots for methemoglobin fraction (FMetHb %) non-survivors compared to survivors (*P* = 0.05). The horizontal bars represent the median (central bar), and the minimum and maximum values (peripheral bands). The dotted line marks the upper reference interval for venous methemoglobin (2.2%).

**Figure 4:**
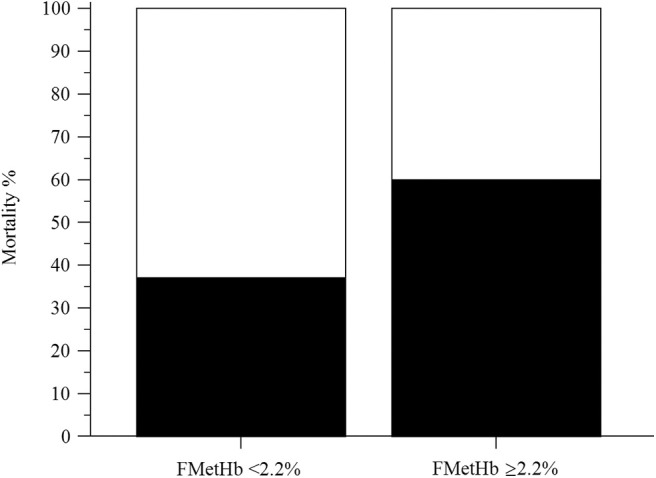
Bar chart with 100% stacked columns showing the percentage of mortality in dogs with methemoglobin fraction (FMetHb %) <2.2% compared to dogs with FMetHb ≥2.2%. The difference was statistically significant (*P* = 0.04).

In the overall population of dogs with sepsis, 24/131 (18%) had a circulating methemoglobin ≥2.2%. The main laboratory findings in dogs with sepsis and septic shock are reported in Table 1. The median methemoglobin fraction was significantly higher in the whole population of septic dogs compared to healthy ones (1.7%, 0.4–3.5% vs. 1.0%, 0.3–2.2%; *P* = 0.0005) (Figure 1). No significant difference was observed in the comparison of methemoglobin values between dogs with sepsis vs. dogs with septic shock (1.8%, 0.4–2.8% vs. 1.5%, 0.4–3.5%; *P* = 0.74), and between survivors vs. non-survivors (1.5%; 0.4–2.8% vs. 1.8%; 0.4–3.5%, *P* = 0.05) (Figures 2, 3). Dogs with an APPLE_fast_ score ≥25 had comparable methemoglobin levels to dogs with an APPLE_fast_ score <25 (1.6%, 0.4–2.8% vs. 1.9%, 0.6–3.5%; *P* = 0.40). Similarly, no significant difference was detected in venous methemoglobin fraction between dogs with and without MODS (1.7%, 0.4–3.5% vs. 1.7%, 0.5–2.8%; *P* = 0.27). The presence of circulating methemoglobin levels above the RI determined in our study was associated with higher frequencies of death in our study population (60.0% vs. 36.8%; *P* = 0.04) (Figure 4), as well as with the occurrence of hepatic dysfunction (37.5% vs. 11.2%; *P* = 0.004) (Table 2). There was a trend for higher frequencies of MODS occurrence in cases with methemoglobin levels ≥2.2%, but this difference did not reach statistical significance (45.8% vs. 25.2%, *P* = 0.07). Despite similar trends for most of the other organ dysfunctions occurring in the study population, no significant difference was noticed in respect to methemoglobin RI (Table 2).

**Table 1:** Descriptive statistics of selected clinical and clinicopathological variables in healthy controls, dogs with sepsis, and dogs with septic shock.

**Variables**	**Healthy**	**Sepsis**	**Septic shock**	**RI**
**CLINICAL DATA**				
Days of hospital stay	NA	3 (0–19)	3 (0–14)	NA
Rectal temperature (°C)	NA	39.2 (35.5–41.6)	38.5 (33.0–41.1)	NA
Pulse rate (bpm)	NA	130 (52–200)	160 (80–235)	NA
Respiratory rate (rpm)	NA	35 (12–108)	38 (16–68)	NA
SBP (mm Hg)	NA	140 (90–199)	83.5 (40–152)	NA
APPLE_fast_ score	NA	25 (13–33)	27 (13–38)	NA
**BLOOD GAS ANALYSIS**				
pH	7.36 (7.23–7.43)	7.35 (7.06–7.55)	7.26 (6.84–7.40)	7.28–7.41
pCO_2_ (mmHg)	41.5 (27.9–43.3)	36.0 (13.7–61.0)	41.7 (17.0–75.5)	31.0–50.5
HCO_3_ (mmol/L)	22.1 (16.4–28.2)	18.2 (6.2–32.8)	17.6 (6.7–29.5)	17.5–25.1
Lactate (mmol/L)	1.3 (0.5–3.0)	2.2 (0.4–7.2)	3.5 (1.3–9.9)	0.2–1.5
FMetHb (%)	1.0 (0.3–2.2)	1.8 (0.4–2.8)	1.5 (0.4–3.5)	0–2.2%
iCa (mmol/L)	1.32 (1.25–1.41)	1.23 (0.85–2.07)	1.20 (0.81–1.44)	1.24–1.41
Sodium (mmol/L)	145 (140–148)	141 (115–173)	141 (118–163)	141–149
Potassium (mmol/L)	4.0 (3.4–4.5)	3.8 (2.6–6.6)	4.0 (2.7–6.1)	3.5–4.5
Chloride (mmol/L)	117 (110–123)	114 (93–141)	113 (91–147)	113–122
**COMPLETE BLOOD COUNT**				
HCT (%)	51.0 (42.9–60.2)	40.0 (18.0–56.5)	42.7 (26.2–68.5)	37.0–55.0
Hemoglobin (gr %)	17.6 (14.9–20.6)	13.3 (3.6–23.3)	14.2 (6.7–20.5)	12.0–18.9
Platelets (x10^3^/mm^3^)	245 (148–355)	252 (23–729)	244 (29–823)	160–500
WBCs (cells/mm^3^)	8,190 (4,200–14,200)	15,920 (380–58,350)	8,845 (910–48,650)	6,000–17,000
**SERUM CHEMISTRY**				
Creatinine (mg/dL)	1.13 (0.74–1.42)	0.94 (0.26–14.80)	1.57 (0.30–11.16)	0.75–1.40
Urea (mg/dL)	33.6 (17.7–48.5)	30.25 (7.6–658.0)	66.97 (15.2–575.2)	17.0–48.0
Total bilirubin (mg/dL)	0.21 (0.09–0.34)	0.23 (0.02–26.60)	0.27 (0.01–7.20)	0.07–0.33
Total protein (g/dL)	6.44 (5.74–7.65)	6.19 (3.64–9.02)	5.63 (2.71–8.97)	5.60–7.30
Albumin (g/dL)	3.29 (2.71–3.91)	2.50 (1.51–4.01)	2.27 (1.08–3.83)	2.75–3.85
CRP (mg/dL)	NA	25.97 (1.95–50.08)	23.80 (1.20–55.70)	0–0.5

*Data are reported as median and (min-max). APPLE_fast_, Canine Acute Patient Physiologic and LaboratoryEvaluation; CRP, C-reactive protein; FMetHb, methemoglobin fraction; HCO_3_, bicarbonate concentration; HCT, hematocrit; Hb, hemoglobin; iCa, ionized calcium; NA, not applicable; pCO_2_, partial pressure of carbon dioxide; RI, reference interval; SBP, systolic blood pressure; WBCs, white blood cells*.

**Table 2:** Selected organ dysfunction and MODS occurrence in septic dogs with circulating methemoglobin fraction ≥2.2% (*n* = 24) vs. <2.2% (*n* = 107).

**Organ dysfunction**	**Frequency for FMeth ≥2.2%**	**N**	**Frequency for FMeth <2.2%**	***N***	***P*-value**
Hepatic	37.5%	9	11.2%	12	**0.004**
Renal	41.7%	10	24.3%	26	0.12
Respiratory	20.8%	5	12.2%	13	0.32
Hemostatic	25.0%	6	15.9%	17	0.37
Cardiocirculatory	16.7%	4	19.6%	21	1.0
MODS	45.8%	11	25.2%	27	0.07

*Significant P-values are bold. FMetHb, methemoglobin fraction; MODS, multi-organ dysfunction syndrome; N, number of cases*.

## Discussion

In this preliminary study, we evaluated venous methemoglobin fraction in dogs with sepsis. Our results partially parallel the human ones: the median methemoglobin fraction was greater in septic dogs compared to controls. However, values of methemoglobin above the RI were rare, and its levels were not able to identify the presence of septic shock or MODS occurrence. Nonetheless, hepatic dysfunction was much more common in patients with methemoglobin fraction ≥2.2% (upper limit of our RI). Similarly, trends in the occurrence of MODS, renal, respiratory and hemostatic dysfunction appeared higher in cases with methemoglobin above or equal to the identified RI (Table 2), but did not reach statistical significance. Redox mechanisms of sepsis have been implicated in mithocondrial and cellular dysfunction (11), and high circulating methemoglobin fraction has been occasionally associated with organ failure in septic humans (9). It is hard, however, to draw conclusions from these preliminary data in dogs, as methemoglobin fraction was mildly elevated in our population, and the number of cases compared in each group was low. Non-survivors had higher values of methemoglobin compared to survivors, although the statistical significance was only borderline. Anyway, septic dogs with a methemoglobin ≥2.2% had significantly lower survival rate compared to the ones with circulating methemoglobin below this threshold. This result demands consideration, as dogs with sepsis, like humans, have circulating methemoglobin fraction, which can be higher than normal, potentially indicating NO overproduction in some of these patients. These data do not seem to be clinically relevant in the majority of cases, as the clinical signs of methemoglobinemia are not usually described in sepsis, and were not reported in our study population. However, subsets of septic patients with circulating methemoglobin values above the RI tend to have a worse prognosis. Whether circulating methemoglobin represents only a marker of the burden of sepsis that goes unnoticed using conventional scores to classify disease severity, or concur itself to worsen sepsis progression, remains a matter of debate that has to be clarified.

There is a limited number of studies investigating NO and its metabolites in canine sepsis. Plasma nitrites and nitrates were higher in septic dogs compared to dogs with non-infectious systemic inflammation and healthy ones, but their diagnostic power was poor, and they lacked any prognostic role (14). Although the relationship between NO metabolites and the development of endotoxic shock is still unclear (12, 13), it seems that iNOS inhibitors might have positive hemodynamic properties and reduce the conventional dose of vasopressors in patients with septic shock (18).

According to experimental and clinical studies, methemoglobin itself is a critical mediator of systemic inflammation and oxidative damage, and may have several propagating effects on sepsis development. Methemoglobin leads to increased availability of iron to pathogens, which may fuel their proliferation (1, 11). In addition, methemoglobin behaves as a highly redox-reactive major damage associated molecular pattern, and through the interaction with the Toll-like receptor type 2 induces apoptosis of neutrophils (19, 20). Finally, methemoglobin activates endothelial cells by stimulation of inflammatory cytokines, leading to further iNOS expression, thus closing a “NO-generating loop” (1, 11). The presence of high fraction of circulating methemoglobin in sepsis has been highlighted by few studies in humans (1–3, 9, 21). Overall, increased fraction of methemoglobin in blood during sepsis seems to be an underdiagnosed and underestimated condition, because usually mild and poorly symptomatic. Selected subsets of critical care patients (e.g., neonates), however, might be exposed to the detrimental consequences of increased methemoglobinemia, which should not be unnoticed (2). Despite the controversial results of the different studies available in the literature, methemoglobin has been suggested to promote sepsis-related complications including inappropriate vasodilation and organ dysfunction (9, 21). Nowadays, methemoglobin concentrations can be easily measured with multiple wavelength co-oximeters or modern co-oximeter blood gas analyzers, that typically measure the optical absorbance of blood at 4 up to 128 different wavelengths and automatically compute the fractional concentrations of the four major hemoglobin species (oxy-, deoxy-, carboxy-, and methemoglobin), as well as the total hemoglobin concentration (22). Given the growing availability of these instruments in clinical practice, in the authors' opinion, methemoglobin evaluation could have a place in the diagnostic work-up of septic patients or when a suspicion of sepsis is aroused. Additionally, methemoglobin role in specific subgroups of septic veterinary patients (e.g., during neonatal or pediatric sepsis, or during bloodstream infection), as well as in different critical care settings, has to be addressed in further studies.

There are some limitations to acknowledge when interpreting our results. Although patients were retrospectively enrolled based on the availability of methemoglobin levels at ICU admission, the general limitations of a retrospective study design were overcome due to the previous prospective inclusion of these dogs in a parent study on sepsis and MODS. Nonetheless, some of the investigated variables (e.g., the APPLE_fast_ scores) were not available for all the included cases. Methemoglobin fraction was recorded at the time of admission only; hence it represents the initial snap-shot of our septic cases, which were indeed affected by a dynamic condition. Serial methemoglobin measurements during hospital stay might have generated different results in terms of groups' comparison. Selected blood characteristics (e.g., lipemia), or hemoglobin-based solutions administered to the patients interfere with co-oximetry readings (22); although methemoglobin values were retrospectively retrieved, the role of such interferences was minimal, as lipemic samples are routinely discarded by our laboratory personnel, and blood substitutes were not used in the enrolled dogs. The population of septic dogs was heterogeneous in terms of sepsis diagnosis, limiting the conclusion on circulating methemoglobin in specific settings of sepsis. In this regard, a preliminary comparison of methemoglobin fraction among subsets of patients distinct for age and underlying disease (e.g., dogs with parvoviral enteritis vs. whole study population) was attempted (data not shown), but the small sample size of the subgroups was considered a potential source of bias. Furthermore, a group of critically ill dogs without sepsis was not available for comparisons, hence the role of methemoglobin as a general biomarker of critical illness regardless the presence of sepsis remains unknown. This was, however, beyond the scope of the present study. Finally, the control group selected for RI generation was small, and not age-matched with our population of septic dogs (data not shown).

To conclude, this is the first study documenting increased circulating methemoglobin fraction in dogs with sepsis compared to healthy dogs, and to assess its value as a prognostic biomarker for this condition. A mild increase in circulating methemoglobin was a common occurrence in dogs with sepsis, but did not differentiate dogs with uncomplicated sepsis from the ones with septic shock. Nevertheless, sepsis can be considered a differential diagnosis of increased methemoglobin fraction in dogs. Circulating methemoglobin levels above normal seems to be associated with higher death frequencies in septic dogs, thus being an additional variable to monitor in the course of this disease. Further prospective studies are needed to fully characterize the clinical and the prognostic significance of methemoglobinemia in animals with sepsis, to understand its role in promoting organ dysfunction, and to better define the subsets of septic patients where its monitoring is warranted.

## Data Availability Statement

The datasets generated for this study are available on request to the corresponding author.

## Ethics Statement

The studies involving animals were reviewed and approved by the Animal Welfare Committee (COBA) of the Alma Mater Studiorum—University of Bologna. Written informed consent was obtained from the owners for the participation of their animals in this study.

## Author Contributions

RT, MG, and FD designed the study, analyzed data, co-wrote, and edited the manuscript. EC, KV, and AF analyzed data and edited the manuscript. All authors contributed to read and approved the final manuscript.

## Conflict of Interest

The authors declare that the research was conducted in the absence of any commercial or financial relationships that could be construed as a potential conflict of interest.

## Abbreviations

APPLE_fast_: Acute Patient Physiologic and Laboratory Evaluation Fast Score
CBC: complete blood count
CRP: C-reactive protein
iNOS: nitric oxide synthase
ICU: intensive care unit
MODS: Multiple Organ Dysfunction Syndrome
NO: nitric oxide
RI: reference interval
SIRS: systemic inflammatory response syndrome.
